# A Systematic Treatise on Comparative Physiology, Introductory to the Physiology of Man

**Published:** 1835-01-01

**Authors:** 


*A Systematic Treatise on Comparative Physiology, introductory to the Physiology of Man. Translated, with Notes, from the German of* FREDERICK TIEDEMANN, Professor of Anatomy and Physiology in the Heidelberg University, *by* JAMES MANBY GULLY, M.D., *and* J. HUNTER LANE, M.D., F.L.S. &c. *Vol. I.* —London and Liverpool, 1834. 8vo. pp. 431.

COMPARATIVE anatomy and physiology are at length beginning to be estimated at their just value. After a slumber of more than two thousand years, they rose up, under the auspices of Hunter and Cuvier, to give a new impulse to the science of life, and to add new lustre to the fame of Aristotle, whose genius was so far in advance of the times in which he lived, that the accumulated knowledge of ages has alone enabled us to understand, and appreciate his labours.

The importance of these sciences to medicine has been generally recognized only within the last few years, and is even now imperfectly understood, if we may judge from the limited number of those who cultivate them.

As a means of determining the functions of organs, comparative anatomy and physiology are invaluable, since they enable us to avoid the numerous fallacies inseparable from all experiments on living animals which are attended with mutilation. In the vast series of organs and functions which living nature displays, we are enabled, by their aid, to trace functions from their earliest dawn to their highest perfection, in conjunction with the organs on which they depend; or to follow them in the descending scale of organization, till they disappear along with their ministering organs. How much more exact and satisfactory is the information derived from this source, than any which can be drawn from anatomical experiments on the living animal! In the one instance, organs are curtailed or withdrawn by the hand of nature; the organism varying its form, and the number or perfection of its functions, yet still presenting an entire and healthy living body: in the other, the removal or lesion of organs by mechanical violence often leaves for our observation only the wreck of an organism, scarcely animated by a fast-ebbing vitality. The method of comparison has also another great advantage: it enables us to reason synthetically as well as analytically. The anatomical experimenter may take away a part, or the whole of an organ, and mark the consequent abolition of function; but he never can expand an organ into increased development, nor add one to the number of those already existing. The observation of nature, however, affords us this power: we see new organs added, and new functions simultaneously called forth; and we find the relation between the two corroborated by the increasing perfection of the function accompanying the fuller development of the organ. As Sir C. Bell has justly remarked, experiment on the living animal is chiefly useful, as affording the hint which leads to discovery; but, in order to place physiological theory on a broad and firm basis, we must have recourse to analogies derived from an extended survey of the varied forms of life.

While, however, comparative physiology, if philosophically pursued, is of infinite utility, it affords the same scope for hasty generalization, which renders all analogical reasoning dangerous in the hands of those who are not careful to keep their imagination in abeyance to their judgment; and it must be confessed, that several highly fantastic and puerile hypotheses have been reared on a comparison of organized beings. We may briefly allude to one first promulgated by Robinet,* and which has found more favour than it deserves at the hands of some distinguished physiologists.

From the ascending series observed in nature, from the simplest to the most complicated forms of living being, it has been surmised that the author of all things, like other unpractised artificers, was obliged to begin how he could, and gradually attained dexterity by experience. It is not much

* Considerations Philosophiques de la Gradation Naturelle des Formes de l'être; ou, les Essais de la Nature qui apprend a faire l'Homme. Paris, 1768. 8vo.

worth while to argue against a doctrine so much opposed to the first principles of ethics; it would not, however, be difficult to turn against its supporters their own kind of reasoning. For example: the human organism is considered by these philosophers as the highest achievement of creative power. Now we find this organism progressively developed *in utero*, from a simple drop of fluid, in which there exists not one of the complicated organs afterwards to be evolved; we find it passing through several stages of development, each resembling, to a certain degree, the organization of some animated being lower in the scale than man; though the analogy be not so complete as those physiologists would have us believe, who regard the development of the human fœtus as a kind of epitome of the whole animal creation. Again, in the development of the human fœtus, there are, from the very beginning, powers at work, mighty enough to build up the majestic frame of man,—causes prospectively adapted to this end; and, if their operation be gradual and progressive, what can we see in this but the wise adaptation of the organism to the several stages of its intra-uterine life, whose regular succession marks the footsteps of infinite power and beneficence, and whose inversion would render the existence of man impossible, or, if possible, absurd? If then it be clear, in this striking instance, that progressive development from more simple to more complex forms of life is the result, not of defective power in the beginning, but of prospective wisdom, looking towards the consummation, why should we take an opposite view of the series which animated nature now presents, or the wrecks of successive worlds unfold? Why should we not rather believe, in accordance with all we can observe of the relation of past and present living beings to the things around them, that each organism is exactly adapted to the state in which it is to live, and that, where organs are not given, it is because such organs would be useless? Such a view of the subject is strongly corroborated by the fact, that, in the organisms which we are accustomed to consider as inferior, whenever high perfection in any one organ is required, that perfection is found to exist. Thus, in the *crinoidea*, a family of radiated animals, which were among the earliest inhabitants of our planet, we find an organ of apprehension, quite as wonderful, and infinitely more complex, than the human hand.* Many other examples might be given; but the argument has been recently and very ably

* For the anatomy of these animals, see Miller's Natural History of the Crinoidea, &c. Bristol, 1821.

handled by Sir Charles Bell, who has one remark, so ingenious and apposite, that we cannot refrain from quoting it.

"We are led to this reflection, that the creation of a living animal, the bestowing of life on a corporeal frame, however simple the structure of that body, is of itself an act of creative power so inconceivably great, that we can have no title to presume that any change in the organization, such as the provision of bones and muscles, or the production of new organs of sense, is a higher effort of that power."*

The only supposition on which the idea of progression in creative power could be reasonably entertained, would be, that the Deity committed the fabrication of the world to inferior intelligences, and made it a sort of school of arts for the improvement of their ingenuity; a notion which might perhaps be grafted on the philosophy of the modern Platonists, but which is quite dissonant from that of the nineteenth century.

But we are physiologists, not theologians; apologizing therefore for the preceding remarks, which, however, we trust, will not be deemed altogether inappropriate as an introduction to our subject, we proceed to examine the volume before us.

This system of comparative physiology exhibits an extended acquaintance with facts and opinions, combined with cautious reasoning, and a candid spirit of inquiry. As this is the first work of the kind that has appeared in the English language, we shall give a short analysis of the contents of the entire volume, commenting upon them as we find occasion.

The first book is occupied with *a Comparison between Living Bodies and Bodies not endued with Life.* If it be true that all the physical sciences blend with each other, it is not less true that there are points at which each of them merges in metaphysics,—at which our progress is arrested by the difficulty of analyzing our own ideas, and affixing to the terms we use a precise and intelligible meaning. The attempts hitherto made to define an organised being, as distinguished from one that is inorganic, afford illustrations of this remark. Tiedemann has wisely abstained from any such attempt, and has employed himself more profitably in ascertaining the cognisable properties of each class of bodies. These are treated of under the heads of *Chemical Composition*, and *Mechanical Texture and Configuration.*

We have only space here for the general conclusions of our author. On the subject of chemical composition he observes,

* Bridgewater Treatise, p. 39.

"The principal result of the comparisons made between the composition of organic bodies and those of inorganic bodies, which comparisons are founded on observations and researches in the chemical composition of these bodies hitherto pursued, is that the former have peculiar matters, which we call organic, for their basis. The changes of composition which take place in bodies endued with life, are not simply the effects of affinities similar to those observed in brute or inorganic bodies; they are the effects of affinities and forces of a special nature. Organic matters are the only ones which exhibit, and for the greater period of time in a particular state of aggregation and form, called organization, the manifestations of activity which we designate by the name of life. They are accumulated at one time in bodies actually living, and life is manifested in them; at other times, they are exterior to living bodies mixed with inorganic matters, and then only capable of living. In this latter state they may return to the domain of living bodies, and into the tide of life, either in the shape of aliments, or, in a direct manner, by the aid of certain circumstances, as happens in spontaneous generation. Purely chemical affinities, or the action of simple chemical forces, appear, in the present state of our planet, to produce no organic combination or matter, such as albumen, gelatin, starch, gluten, &c.: at least, we possess no facts which go to support the contrary opinion. None but organic bodies themselves are capable of introducing inorganic matters into organic combinations, of which respiration in particular, and the nutrition of plants, are examples." (P. 12.)

A general comparison of the mechanical properties of the two classes of bodies is contained in the following passage.

"From the parallel which has been established between the form and aggregation of organic and those of inorganic bodies, essential differences are collected. All organic bodies have a regular form, terminated by undulating lines and surfaces, which are not flat. They all proceed from an assemblage of heterogeneous parts, both liquid and solid, having a peculiar mode of arrangement and distribution, and connected so as to produce an harmonious whole; in other words, engaged in a reciprocity of action necessary to the preservation of the individual. The form and aggregation sway each other mutually; the destruction of one leads to that of the other. All organized bodies preserve their form and aggregation by virtue of an internal activity under the influence of external circumstances, and amid incessant changes in their material substance, or their composition. They are developed from each other, produce themselves, are formed and maintained by their own activity, are subject to regular changes, and enjoy a certain durability.

"These bodies thus constitute separate beings, whose various parts, with their different qualities, have a configuration and an aggregation of such a nature, that unity, harmony, concurrence of actions to a common end, the preservation of the individual and of the species, may, and in fact do, follow as results. They are relatively more perfect than inorganic bodies. This superiority of relative perfection is exhibited by the greater number of different parts and matters entering into their composition, as also by the more intimate connexion and more exact reciprocity of action existing between all these parts and matters, so that we cannot but recognise a train of coincidences tending to one end, or to unity of end." (P. 26.)

The origin of organic bodies has given rise to much interesting speculation; we need make no apology for introducing a long extract, which contains a very judicious review of the opinions that have been entertained on this curious subject.

"The solution of this problem passes the limit of our experience. Should we, however, wish to hazard an answer to it, we fall into the waste of conjecture, and are forced to erect hypotheses, which are but probable, and not at all certain. We suppose that organized bodies have existed in our planet from its commencement; or else we admit that organic matters and living bodies have been produced, under certain circumstances, together with the elements and inorganic matters, by the action of general physical causes; or, lastly, we conjecture that the substance of living bodies was primitively contained in water, as primitive organic matter, having the property of taking on the organic form; that it gave origin to organic bodies of very simple and varied kinds in consequence of circumstances, and that these bodies have passed successively to more complicated forms, until at length the generative organs and their manifestations of activity having appeared in them, they were endowed with the faculty of preserving themselves in a continuous manner, by means of generation, as separate species.

"Geology is opposed to the first hypothesis of the existence, in our planet, of living bodies from the first moments of its creation. Fossils are found only in the exterior crust, that is to say, in the superficial layers of the earth, the formation which is most recent, whilst there are none at all in the primitive earths. Consequently, there was a time when no living being existed on the globe. Even supposing we admitted this hypothesis, we should still leave untouched the question, how living bodies were formed, inasmuch as we could say nothing concerning the mode of origin of our planet and of the bodies which constitute it. In reference to this question, it matters little whether we declare for Vulcanism or Neptunism, since the geologists are under the necessity of leaving the origin of fire and water without explanation, and the biologist is still less able to pronounce any opinion on that of living bodies.

"The difficulties which occur in the second hypothesis, of the dependence of the production of organic matters and living bodies on the action of general physical forces, are, that we are actually in want of facts which would authorise us to conclude analogically that organic matters and living bodies can proceed from inorganic matters, never having observed any thing similar, at least, up to this day. Far from this being the case, living bodies are unable to produce, with inorganic substances, the greater number of the materials which enter into their composition, and for such end they require the matter of other organic bodies, which they introduce into themselves. Plants are nourished principally by the remains of dead vegetables or animals: animals likewise preserve their existence by means of vegetables, and even of other animals.

"The most probable hypothesis is the third, viz. that the substance of organic bodies existed primitively in water, as matter of a particular kind, and that it was there endowed with the plastic faculty, that is to say, with the power of acquiring, by degrees, different simple forms of living bodies, with the concurrence of the general influences of light, heat, and perhaps also of electricity, &c., and of then passing from the simple forms to other more complicated, varying in proportion to the modification occurring in the external influences, until the point when each species acquired duration by the production and manifestation of activity of the genital organs. Although we cannot here also answer the question, whence came the water and the organic matter which it contained, yet this hypothesis is the one which accords best with the facts with which geology has latterly been enriched. In fact, we find no organized bodies belonging to what is called the primitive world in the strata of earths which modern geologists consider as the products of fire or of Vulcanism. They are only observed in the upper layers of the earth, in those of the latest formation, and in the soils which have evidently been precipitated in the midst of the waters. Aquatic animals existed before terrestrial animals. An argument which favours the hypothesis according to which the organic kingdom has been gradually developed and elevated from simple to more complicated forms, is drawn from the fact, that we meet with remains of organic bodies belonging to the most simple species in the secondary and more ancient soils, whilst the most recent strata of the earth contain the remains of more complicated living bodies. The soils which rest directly on primitive rocks present fragments of corals, radiated animals, and shells. It is only after these that remains of vertebrated animals, fishes, reptiles, and cetacea, are found in the water. Fossil bones of oviparous animals exist in the deep strata of the earth, whereas the viviparous mammiferæ are met with in the superficial layers. We observe the same in the organic complication of vegetables whose remains are contained in the different layers of the earth. Impressions of cotyledonous plants, especially of ferns, are the first vegetable traces met with in the deep seated strata. Then come the remains of monocotyledonous plants, of aborescent gramina, of palms, &c., and finally, those of the coniferæ, and other dicotyledonous plants.

"There have not yet been found any fossils belonging to apes or man, whose organization has reached the highest degree of complication and development. We may therefore admit, with great probability, that apes and men are the last and the newest products of our planet." (P. 13.)

The translators have here inserted the following ingenious note.

"An additional argument in favour of this hypothesis, is the fact, that whenever animal matter shall have lost that power which gave and maintained it in a higher degree of complication in form and functions, no matter how high this degree, it invariably returns to the most simple forms. The noble human form, after the cessation of the functions, possesses only sufficient plasticity to take on the shape of the lowest insects and worms. The same applies to the kingly lion of the forest, and the soaring eagle. In fact, the matter composing each of these, after death, is in the same state as the matter which is described by Tiedemann as possessing merely the aptitude for life, and therefore taking on only the most simple form. Again, that external circumstances modify structure, is very well ascertained. The absence of light generally causes a mother to produce a deformed child, as Edwards observed in females confined in dungeons; whilst tadpoles, preserved from the light, became huge tadpoles instead of frogs. Natives of different climes have different parts of their organization prominently in action: the muscular system, for instance, is much more developed in cold than warm climates; on the other hand, natives of the tropics are from birth more excitable than those of northern parts of the globe; in other words, the animal nervous apparatus is more developed. In a pure hypothesis it is not expected that the modus operandi of the circumstances to which Tiedemann alludes should be explained; collateral evidence is certainly in favour of it." (P. 16.)

The remark is original and apposite.

"Another circumstance," continues Professor Tiedemann, "favourable to the hypothesis of the gradual development of organic bodies, from the most simple to the most complicated, is, that all those bodies, as well vegetables as animals, to this day appear in a simple form, at the period of generation, or when they proceed from the germ, and that it is only by degrees they acquire the most complex form peculiar to such species. To commence in a very simple manner, and to rise thence to the complicated, is the general character of every thing that has life, as well of individuals as of the entire of the organic kingdom.

"These reasons, coupled with the fact that, after the extinction of the life of individuals, the materials of organized bodies are reduced to the most simple organic forms by the action of what is called spontaneous generation, oblige us to admit a primitive organic matter extended on the surface, or in the crust and waters of our planet, concerning the first origin of which matter it is as possible for us to certify any thing as on that of the planet itself. This organic matter, with its different organic modifications, considered as matters of a peculiar species, sometimes is seen active and living in the individuals of vegetable and animal species actually existing, under conditions and in the midst of phenomena, the recital of which will be made hereafter; at other times remains merely capable of enjoying life, and endued with the faculty of taking on, in certain circumstances, the most simple organic forms, whenever it has been withdrawn from the composition of living bodies.

"Several naturalists, particularly Buffon and Needham, have allowed the existence of a matter peculiar to living bodies. G. R. Treviranus concludes from his researches on life—

"1. That there is in nature a matter which is ever moving, by which all living beings, from the byssus to the palm, and from the infusoria animalculæ to the sea-monster, possess life, and which, though immutable in its essence, is, notwithstanding, variable in its form, and is incessantly changing it.

"2. That this matter is deprived of form in itself, but nevertheless ready to take that of life; that it maintains a determinate form under the influence of external causes; that it only continues in that form so long as these causes are active, and that it takes another so soon as new causes influence it.

"3. That the matter capable of life, and the living principle, exist reciprocally, and that death is only a passage of certain forms of this matter to certain others." (P. 16.)

It may be interesting to compare the view here taken of living matter by Treviranus with that of John Hunter.

"Animal matter, then," says the latter author, "is the result of a peculiar combination of other matter; and this combination, or modification of common matter, may be endowed with the living principle or not, the living principle not depending on that modification of matter which renders it animal matter; for this modification, which is peculiar to animal matter, and which constitutes it, may remain when the living principle is gone. Life is therefore something superadded to matter thus modified, or it is such an arrangement of the most minute particles of this modification as life may arise out of it, which arrangement may be the principle of life; and, if this arrangement be destroyed, the principle of life is also destroyed, and the part becomes dead animal matter; and, though it has suffered this alteration in its arrangement, and the loss of this principle, yet its modification, as far as our senses can discover, remains the same."*

The philosophical caution of this opinion of Hunter's cannot be too much admired.

* Parkinson's Hunterian Reminiscences, p. 2.

The hypothesis maintained by Treviranus may also be advantageously compared with that of Aristotle, who taught that a plastic energy exists throughout the universe, which causes matter to assume the forms of life: ἡτε ἐν φυτοις, και ζωοις, και δια παντων διηκουσα, εμψυχος τε και γονιμος ὀυσια,—"pervading plants, and animals, and all things,—a living and generative nature."*

He appears to have considered all *activity* as *life;* for he tells us that the heavens, (meaning thereby the universe,) are animated;† and he ascribes their activities to a portion of the soul of the universe, just as he ascribes the unconscious actions of the animal body to a portion of its soul.‡

The question how far all activity is life need not be argued, because it is one merely of words; but the fact that ordinary matter is never observed to take on the forms of organisation, appears to justify the conclusion of Treviranus and others, that the capacity of doing so is confined to a particular kind of matter. In separating the plastic power from the matter of which it may possibly be only a property, Aristotle falls into an error, exceedingly common with him and all the Greek philosophers,—the disposition to mistake properties for substances, to make entities of attributes. On the other hand, Treviranus is incautious in asserting absolutely that the matter capable of life and the living principle exist reciprocally: life may be a property of matter, or it may be something superadded to matter,—we know not which.

In the infancy of geology, the Aristotelian doctrine of plasticity gave birth to a very curious notion. The organic impressions found in rocks were considered, not as the actual remains of living beings, but as the productions of the *vis plastica,* vainly labouring in the bowels of the earth to evolve the forms of life: nature, hemmed-in and oppressed by unfavourable circumstances, could produce nothing but these stony abortions. This opinion, though often controverted, pervaded the writings of geologists, from Albertus Magnus§ to Dr. Plott,|| the latest author of note who defended it.

The idea is one of the wildest in the romance of science;

* Lib. De Mundo, c. iv., ed. Du Val.
† De Cœlo, lib. ii. c. 2, 12, ed. Du Val.
‡ Vide Cudworth's Intellectual System of the Universe, book i. c. 3.
§ De Mineralibus et Rebus Metallicis, lib. i., tract ii., c. 8. Ed. Colon. 12mo. 1569.
|| Natural History of Oxfordshire, c. v. Folio. Oxford, 1705.

a subject on which an entertaining, and perhaps not uninstructive, work might be composed.

The subject of the second section of this book is a *Parallel between the Manifestations of Activity of Organic and those of Inorganic Bodies.*

The first chapter treats of the manifestations of activity common to organic and inorganic bodies, and their modifications in the former.

The causes of activity in organic bodies may all be reduced to repulsion and attraction: the first includes impenetrability and extension; the second, mechanical attraction, gravity, cohesion, and chemical affinity. These properties are common to inorganic and organic bodies, but in the latter they become modified by *life*, which always influences, and sometimes appears to antagonize them.

The second chapter is on the manifestations of activity proper to organic bodies.

All organic bodies are in a state of continual activity, and undergoing perpetual changes. Some of their actions and mutations take place uninterruptedly during the whole life of the individual; there are others which present themselves only once, and some that occur only at certain periods. In the first class, nutrition holds a prominent place; and, on the whole, the functions by which a living individual maintains itself as such, are the following:

"1. Taking from the external world liquids and solids, entitled aliments, which is performed by absorption, or by particular movements.

"2. Absorption of gaseous substances from the media which surround them, and expulsion of their materials in the same form, to wit, respiration.

"3. Conversion of the food, or gaseous bodies, which they have absorbed, into a mass resembling that of their own humours, —assimilation.

"4. Movement of the humours between the interstices of the solid parts,—the circulation.

"5. Conversion of the humours into solids, or combinations of these humours with the solid parts, and preservation of the properties of the latter,—nutrition properly so called.

"6. Lastly, preparation of particular liquids, at the expense of their humours,—secretion." (P. 31.)

The second class of vital activities, which come into play only once during the existence of the individual, are those connected with the origin, the development, and the periods of life or of age. The consideration of the origin of life leads the author to the theory of generation. The kinds of generation are three.

1. *Spontaneous generation,* (generatio æquivoca, spontanea, heterogenea, primitiva,) as observed in the products of putrefaction. A simple modification of this is found in the formation of the *entozoa.* On these our author remarks,

"The reasons alleged by Pallas, Müller, Werner, Bloch, Goeze, Braun, and especially G. R. Treviranus, Rudolphi, and Bremser, forbid our admitting that they come from without the animal, since they are not found out of it, nor in the earth, nor in plants, in which they cannot exist, and since they are met with sometimes in organs, such as the eye, the brain, the muscles, and others, which have no communication whatever with the external parts. We must, therefore, with these naturalists, regard them either as the products of non-assimilated elements, or as morbid productions, which, in certain circumstances, are formed in the humours or the parenchyma of the organs. However, many of them, being once formed, are provided with genital organs, and are reproduced by generation properly so called.

"The results of observations and researches on this subject equally impose on us the necessity of admitting the axiom established by G. R. Treviranus,—that there exists a matter peculiar to organic bodies, capable of life, and having the property of taking on certain forms, at the same time that it acquires a particular mode of action. The manner in which this property is manifested in the actual development of form, is dependent on the conflict and reciprocity of action existing between organic matter and external or physical influences, according to which circumstances it takes either an animal or vegetable form." (P. 40.)

2. *Generation by division or scission of an organ into new individuals,* (generatio fissipara,) which occurs only in very simple organised bodies, composed of one homogeneous substance; as in some infusoria, in confervæ, and sometimes in fresh-water polypi; in which latter, however, it scarcely ever happens, otherwise than accidentally, and under particular circumstances.

3. *Generation by germs.* When organic beings are multiplied by means of parts separated from their bodies, to form new individuals, the parts thus produced are called *germs.* Propagation by germs is divided into that by *shoots* or *sprouts,* and that by *reproductive corpuscules.*

In the propagation by shoots or sprouts, the germ is elevated on the surface of the body producing it, in the form of a swelling, which gradually increases in size, and is finally detached, to constitute a separate individual. This mode of propagation is exemplified in the armed polypi, vorticellæ, corals, &c. In plants, it is only found in conjunction with other modes; thus, many plants which produce seed also multiply by creeping roots, shoots, and twigs.

In the mode of generation by reproductive corpuscules, globules or grains are emitted, from which new individuals proceed.

*a.* The production of these corpuscules sometimes occurs only at a particular part of the parent organism, and is accomplished by special organs, which may be regarded as the first rudiments of the female organs of generation. In the *gorgonia, madrepore, sertularia, red coral,* &c., there is formed, in the central substance of the polypus, and not far from its arms, a small membranous sac, containing the corpuscules, which, being detached, bursts, and discharges the corpuscules. The organ which prepares the corpuscules is annually formed, and thrown off, together with its contents. In other instances, the corpuscules are enclosed in tubes or bladders, as in the *medusæ,* or disposed in a fan-like manner, as in the *actiniæ.* Some vegetables are found to propagate by similar collections of germs, to which botanists have assigned different names; and a number of phœnogamic plants, provided with proper sexual organs, multiply also, independently of these, by knots, tubercles, bulbs, and buds; thus combining the two modes of generation.

*b.* At other times the corpuscules are diffused through the whole organism, and developed in several places; as in certain zoophytes, and in some acotyledonous plants, as *confervæ, ulvæ, fuci,* &c.

4. *Generation by two sexes,* as in the mammalia, birds, reptiles, fishes, crustacea, insects, most of the mollusca and annelidæ, and some of the entozoa.

A third class of vital activities was enumerated, which present themselves only occasionally, or at certain periods.

To this class are referred those diurnal changes in living bodies which are connected with the earth's movement round the sun, and other external circumstances, as sleeping and waking in animals, and the phenomena indicative of corresponding states of repose and activity in many plants: also those manifestations of activity and development which occur at longer intervals; as, in animals, all the phenomena connected with procreation,—the renewal of hair, feathers, skin, scales, antlers, &c.,—hybernation,—migration; in vegetables, the annual changes evinced in germination, the evolution of leaves and flowers, fecundation, the fall of the fruit, the decay of the leaf. These periodical changes arise from the action of light, heat, and other external agents, on living bodies, which varies according to the situation of our planet in reference to the sun.

Among the occasional manifestations of vital activity are to be reckoned the restoration of continuity in divided parts, and the regeneration of those that have been lost. These phenomena are familiar both in the animal and vegetable kingdom.

The author's remark, that regeneration of parts in warm-blooded animals is confined to the epidermoid textures, is not strictly accurate: the translators correct it in a note, in which allusion is made to recorded instances of regeneration of the glans penis and phalanx of the finger. It is added,

"Can we consider the growth of a third set of teeth, in old age, as a regeneration of lost parts, since two sets only are usual? also of their sockets? Some instances of this are quoted by Dr. Mason Good, Study of Medicine, vol. 1, page 55, from the German Ephemerides, Medical Commentaries, &c." (P. 441.)

The second book contains *a Comparison of Animals with Vegetables.*

In the first section their *material composition* is considered, under the heads of chemical composition, external configuration, and aggregation, or intimate structure.

We could have wished to have extracted the chapter on external configuration entire, but we have not space; and, as it does not admit of condensation, we must be content with recommending it to the attention of the reader.

The third chapter, on intimate structure, involves an excellent epitome of general anatomy, the more valuable for including that of vegetables; but it will not be expected that we should enter into such a subject here.

The second section compares the *manifestations of activity* of plants and animals. These are considered under three divisions, treating, 1, of the functions of nutrition; 2, of the evolution of imponderable matters; 3, of the movements.

The functions of nutrition are illustrated under the head of aliments,—of the reception of aliments by absorption,—of the reception of aliments by the buccal aperture,—of the assimilation of aliment in the first passages,—of respiration,—of the movement of the nutritive fluid,—of nutrition,—of the secretion of humours.

Taking the eight chapters devoted to these subjects in the mass, (for it would be impossible to condense them within the limits of a review,) we shall merely offer a few excerpta, as specimens of the author's manner, and inducements to the reader to peruse the entire work.

From the chapter on respiration we may quote the author's summary view of this process, and its uses in the vegetable economy.

"If we compress into a small space the phenomena of the respiration of plants, we see that they consist in the exhalation of water in the vaporous form, and of oxygen gas during the day, under the influence of the sun's light. The water comes from the sap which the roots send, by the sap-vessels, into the parenchyma of the leaves, and from the moisture these absorb in the night-time. The primary effect of the evaporation of this water is the condensation of the organic matters contained in the sap. The oxygen gas is in great part taken from the carbonic acid absorbed during the day, and from the water impregnated with this gas, which the roots bring from the soil, together with the organic matters; these are the sources to which Senebier, Woodhouse, and Saussure assign it. Possibly, it also partly arises from the oxigenated organic combinations contained in the sap, that is, from the acetic acid, the sugar, and the matter analogous to gum. It is not certain that the water of the sap is decomposed by the respiration of plants, as Berthollet and Thomson have supposed, and that a part of the oxygen proceeds thence; Saussure rejects this decomposition as by no means probable. The exhalation of oxygen increases the proportion of carbon relatively to the other elements of the sap, just as its absolute quantity becomes greater by the absorption of the carbon contained in the carbonic acid of the atmosphere. In support of this hypothesis, the experiments of Chaptal, Hassenfratz, and Senebier, may be quoted, from which it follows, that plants which have increased in the shade contain much less carbon than others which have been exposed to the light." (P. 119.)

"The acts of respiration which living leaves execute under the influence of light, are of the highest importance to the life of plants. If plants be deprived of their leaves, or if they lose them by cold or the destructiveness of insects, their nutrition and growth are stopped, the development of the flowers, the act of fecundation of the fruit and seeds, cease to occur, and the already formed fruit does not ripen. It is true, that perennial plants then shoot out new leaves, because the buds which should not open until the year after are developed; but this loss does not on this account less frequently cause the death of vegetables.

"If we inquire in what respect respiration is necessary to the life of plants, we can find no other use for it than to produce the nutritive juice, properly so called, or the cambium, from the sap sucked by the roots. The sap, which reaches the leaves colourless, not coagulable, without globules, and composed of water holding in solution carbonic and acetic acids, a gummy saccharine matter and divers salts, is in them converted into a greenish liquid, partly coagulable and filled with globules, which the nutritive vessels return into the trunk of the plant, where it serves for the proper nutrition, as also to the formation, the development, and growth of the parts. It is from this liquid that, in perennial plants, the matter necessary to the production of new ligneous and cortical layers is deposited; it is this which furnishes the materials of which new shoots are formed.

"The juice expressed from the leaves contains the green fecula, which is precipitated as a sediment. In this fecula are perceived green grains or globules, which do not exist in the sap. From the experiments of Rouelle, Einhof, Proust, Vauquelin, Pelletier, and Caventou, it follows that it is composed of a green resinous matter, soluble in alcohol and ether, and combustible, called chlorophylle, of starch, or matter-like gluten, and vegetable albumen. When the juice is heated, it partly coagulates in flakes, and acids precipitate it. Senebier and Gough clearly demonstrate that the green colour of plants depends on respiration, subservient to the influence of light. The conversion of the matters contained in the sap, of the carbonic acid suspended in water, of the acetic acid, of the sugar, and the gum, into more compound organic combinations, such as they exist in the green fecula, are to be considered as an effect of respiration, for which no satisfactory theory has yet been given. From the facts hitherto obtained concerning respiration, the following is the least strained explanation of it. The matters existing in the sap, the acetic acid, and particularly the gummy saccharine principle, are organic combinations of an inferior kind, containing a vast quantity of oxygen relative to the carbon. Again, in the fecula are found starch, a substance approaching to gluten, and albumen, in the composition of which matters less oxygen enters in proportion to the carbon. It is precisely these changes in the respective proportions of the two elements, which seems to be the result of respiration, since the absorption of carbonic acid from the air augments the mass of carbon, either absolutely or relatively to the oxygen, and the quantity of the latter is perhaps diminished by exhalation. From this it follows, that the organic combinations of a lower degree that exist in the sap are converted into others of a higher degree, which are found in the green fecula. Lastly, in regard to the azote contained in the glutinous matter and albumen of this fecula, it is probably taken from the humours, and is already existing in the sap, wherein some chemists have found an azotized substance. With the formation of organic combinations of a higher degree, which accompanies respiration, seems also to be connected the first appearance of the organic materials of aggregation or globules." (P. 121.)

All the green parts of plants participate in the function of the leaves; while other parts, as the roots, flowers, and fruits, effect chemical changes in the atmosphere, different from those produced by the leaves.

" Roots that have recently been dug from the soil, when placed in a receiver full of moist atmospheric air, from which the stalk and flowers project, and the ends of which alone are immersed in water, absorb oxygen, and exhale a little carbonic acid gas, during the day, according to the experiments of Th. de Saussure. They therefore act like the leaves during the night. When he introduced nitrogen, hydrogen, or carbonic acid gas, into the receiver containing the roots, the plants soon perished.

" The action of the flowers on the atmosphere likewise differs from that which the leaves exert. Th. de Saussure found, in his experiments, that all, even those of aquatic plants, absorb oxygen gas, and that they are not developed in media deprived of this gas. They faded in a vacuum and in nitrogen gas. When a flower is placed under a receiver full of atmospheric air, and stopped by a bath of quicksilver, the quantity of air is but slightly diminished, or even is not at all diminished, so long as there remains any oxygen gas. The flower absorbs the oxygen, and replaces it by a nearly equal quantity of carbonic acid gas. The operation is accelerated by the influence of solar light and heat, whereas it is much more slow in the shade. In general, equal weights of flowers produce more carbonic acid gas than green leaves disengage in darkness, in the same space of time. The absorption of oxygen gas, and the production of carbonic acid gas, take place chiefly by the genital organs. Formerly Saussure supposed, and Grischow also thought he remarked, that flowers exhale nitrogen; but, in his later experiments, he was convinced they gave out neither azotic nor hydrogen gas.

" Regarding the changes which the fruit occasions in the atmospheric air, Th. de Saussure found green fruit determine similar ones to those produced by the leaves. Exposed to the air, they absorb, according to him, carbonic acid gas and exhale oxygen, in smaller quantity, however, and less freely, as they approach the period of ripeness. Berard, again, assures us, that he remarked, in his experiments on the ripening of fruit, that green fruits, raspberries, pears, apples, apricots, figs, cherries, gooseberries, grapes, &c. do not act like the leaves at any period of their growth, under the influence of solar light; that they do not absorb any carbonic acid gas, nor do they exhale oxygen. He maintains that their sole action on the atmosphere, as well in light as shade, consists in the absorption of oxygen and the exhalation of carbonic acid. This contradiction determined Saussure to undertake fresh experiments, and he has shown that green fruits, cherries, plums, pears, and grapes disengage oxygen, and absorb carbonic acid, in the solar light, as well in air containing carbonic acid as in water holding this acid; that, on the other hand, in darkness, they absorb oxygen and exhale carbonic acid gas, and that consequently they act on the air in the same manner as the leaves, although in a less powerful degree. If their growth go on tardily, they destroy the purity of the air under any circumstances, but less in light than in darkness. Lastly, he thought he found that, in the immature state, and at the time they began to get sour, they also absorb a part of the oxygen of the air, which might consequently contribute to the development of their acidity." (P. 123.)

Under the head of Nutrition will be found some philosophical observations on the plastic power of living bodies. Our author strongly and successfully maintains the doctrine of a vital principle, against the iatro-chemists and iatro-mechanicians, whose hypotheses, several times exploded and revived, have been blended into one, which now prevails to a considerable extent on the continent of Europe. The doctrine, we think, can hardly fail to be again, and finally discarded, as one equally opposed to the facts of science, and to the common sense of mankind.

The second division treats of the evolution of imponderable matters.

The functions of life are, in certain instances, attended with those phenomena which some philosophers believe to arise from the movements of imponderable matter, and which others attribute to particular modes of activity in common matter. Such are the production of heat, light, and electricity.

In the chapter on Vital Heat, the author has occupied himself more with facts than theories,—and he is right: for none of the theories hitherto promulgated explain the phenomena. The following is a judicious summary of what is known on the subject of animal and vegetable heat.

"The only point that can be regarded as placed beyond doubt is, that the evolution of heat is a vital act which depends immediately on the process of nutrition, the conditional and preservative cause of life. The taking of alimentary matters, and their assimilation by digestion and respiration, the circulation of the fluids, nutrition, and secretion, the renewal of materials that accompanies the exercise of life, and the incessant changes of composition in the solids and liquids, all which are under the influence of the nerves, also act a part in the production of heat; and it is erroneous to seek the cause of it in any one of these acts only. The intensity of the evolution of heat, and the property of maintaining itself at a certain temperature proper to each species, are, in animals, in direct ratio with the composition of their organization, and with the sum and intensity of their manifestations of activity. Birds and mammifera, which take aliment at the shortest intervals, which digest with the greatest rapidity, which consume the most oxygen, and give out the most carbonic acid,—whose circulation is most rapid and energetic, which exhibit the greatest pertinacity in their movements, in which we observe the strongest effects of the nervous system, which secrete the greatest quantity of diversified humours, and in which, in short, all the phenomena proclaim that the renewal of matters takes place in the most speedy manner; these have the highest degree of heat, and are able to maintain it with the greatest uniformity at the temperature proper to each of them. Amphibia, fishes, insects, mollusca, and worms, whose structure is less complex, whose vital phenomena exhibit less diversity, and in which the above-named factions of life have less intensity, have also a lower degree of heat, are more subject to variation in their temperature, and have their faculty of generating caloric confined to smaller limits.

"Further, the generation of heat resulting from the renewal of matter, and the changes of composition ever accompanying life, varies in animals, within certain limits, according to the development, the periods of age, the nature of aliment, and the manner of performing digestion, according to the respiration, the circulation of the blood and the nervous influence, the seasons, even according to the periods of the day, during waking and sleeping, according to the external stimuli that affect animals, and finally, according to diseases, medicinal remedies, and poisons. The proofs in support of this assertion shall be given when we treat of the heat of man.

"In plants, an evolution of heat seems to occur, though only to a small degree, during the acts of respiraton, nutrition, and secretion, as also during fecundation and germination. But vegetables do not appear to undergo continual changes in consequence of their internal activity, in their solids when once formed, as is observed in animals, in which the matter of the different tissues is incessantly changing; neither do they execute voluntary movements, nor does the entire group of nervous functions belong to them, so that they lack the chief sources of the generation of heat." (P. 247.)

It has been thought that Crawford's theory of animal heat derives great support from the fact, that, throughout all classes of animals, the more the lungs, by the extent and perfection of their organization, favour the chemical changes in the blood, the higher is the temperature of the animal, compared with that of the medium it lives in.* The observation we believe to be just; but we suspect that it is so, only because the higher organization of the lungs is connected with a more perfect development of the whole apparatus of life; and hence, that Professor Tiedemann's estimate of our knowledge of animal heat is correct: it amounts briefly to this,—the greater the activity of the general functions of life, the higher is the temperature of the animal.

The chapter on the Evolution of Light contains a sum-

* Bostock's Physiology, vol. ii. p. 275.

mary of all that is known on phosphorescence. This phenomenon, familiar as occurring in dead vegetable matter, is also stated to have been observed in some living plants, which, as well as the author's observations, are fully enumerated.

"The causes of phosphorescence in vegetables are not yet ascertained. Pultney and Volta regarded it as an electric phenomenon, produced by the idio-electric pollen. But against this may be advanced the fact of Haggren having seen the light proceed from the petals and not from the filaments of the stamina. Probably it is owing, if it occurs at all, to the emanation of a combustible matter, perhaps a volatile oil, which enters into a kind of combustion under the influence of the air. The *Dictamnus albus* is said to spread around it, during the warm summer evenings, an atmosphere that takes fire on the approach of a candle, and gives a brilliant blue flame. The phosphorescence of cryptogamia appears likewise to be owing to a slow combustion, if we may judge from the foregoing experiments." (P. 257.)

The phosphorescence of living animals is also amply illustrated. On the curious subject of the luminous appearance of the sea, the translators give a note, which condenses all that is known on the subject.

The chapter on the Electrical Phenomena of Living Bodies is no less learned and judicious than the rest of the work, and the anatomy of several electrical fishes is given with accuracy and minuteness.

The third division treats of the movements of organic bodies. In the first chapter, the movements of animals are referred, 1, to those of muscles; 2, of the cellular and other tissues; 3, of globules, or organic molecules; 4, movements of turgescence; 5, movements of the nerves. The second chapter treats of the various movements of vegetables; and the third, of the causes and powers which determine the movements of living bodies.

This division involves a review of many well-known physiological theories, and brings us back to the beaten paths of the science, over which the author diffuses a light, derived from the vast range of natural knowledge contained in the preceding portions of the volume. It is not necessary that we should enter into these subjects: we trust enough has been said to convince the reader of the great importance and admirable execution of the work.

To the translators the profession is much indebted for the introduction of such a book into English literature. Their version of the author is correct and perspicuous; and the sound judgment and accurate information evinced in the notes, induces us to regret that their comments have not been more extended.

It is scarcely worth while to allude to typographical errors; but we may just suggest that the correction of not a few will materially improve the appearance of the second edition,—which we hope will speedily be called for, as the work is one which ought to find immediate access to the library of every advanced student of physiology.

